# Comparison of Unilateral Middle Cerebral Artery and Bilateral Middle Cerebral Artery Monitoring for Right-to-Left Shunt Detection by Contrast-Enhanced Transcranial Doppler

**DOI:** 10.3389/fneur.2022.891060

**Published:** 2022-06-02

**Authors:** Wenjie Zhang, Le Yang, Shuli Wang, Lin Wang, Haixian Li, Keshi Yang

**Affiliations:** ^1^Department of Transcranial Doppler Ultrasound, Liaocheng People's Hospital, Liaocheng, China; ^2^Department of Orthopaedics, The Second People's Hospital of Liaocheng, Liaocheng, China

**Keywords:** transcranial Doppler (TCD), right-to-left cardiac shunt, middle cerebral artery (MCA), microbubble (MB), patent foramen ovale

## Abstract

**Introduction:**

Contrast-enhanced transcranial Doppler (c-TCD) is a noninvasive test with high sensitivity for the detection of a right-to-left shunt (RLS). Currently, there are no reports on the outcomes of unilateral versus bilateral middle cerebral artery (MCA) monitoring. This study compared the positivity rate of bilateral MCA monitoring with unilateral MCA monitoring for RLS using c-TCD.

**Methods:**

We enrolled 239 patients (86 women and 153 men) with a mean age of 48.54 ± 13.25 years (range, 14–79 years), who underwent c-TCD examination in the Department of Transcranial Doppler Ultrasound of our hospital between February 2018 and February 2021, due to suspicion of RLS. Bilateral MCA monitoring of 239 patients was performed using dual-channel and dual-depth c-TCD. The positive rate and shunt classification of RLS were calculated for left, right, and bilateral MCA monitoring. The differences in RLS detected by c-TCD monitoring of the left, right and bilateral MCA were compared.

**Results:**

In the left middle cerebral artery (LMCA) monitoring, 35.56% (85 of 239) had a positive RLS result, 38 cases were permanent (44.70%), and 47 cases were latent (55.30%). In the right middle cerebral artery (RMCA) monitoring, 36.82% (88 of 239) had a positive RLS result, 38 cases were permanent (43.18%), and 50 cases were latent (56.82%). In the bilateral MCA group, 43.09% (103 of 239) had a positive RLS result, 50 cases were permanent (48.54%) and 53 were latent (51.46%). The positive rate of bilateral MCA monitoring was higher than that of LMCA and RMCA (43.09, 35.56, and 36.82%, respectively), and the difference was not statistically significant (*P* = 0.193). The positive rate of bilateral MCA monitoring was higher than that of LMCA and RMCA for mild and moderate shunts, but the difference was not statistically significant. The positive rate of bilateral MCA monitoring was equal to that of RMCA, but higher than that of LMCA, with no statistical significance. LMCA monitoring revealed 85 patients with RLS. The sensitivity was 82.52% (85/103). The specificity was 100%. The RMCA monitoring results showed 88 cases with RLS. The sensitivity was 85.44% (88/103). The specificity was 100%.

**Conclusions:**

There was no significant difference in the RLS detection rate between unilateral and bilateral MCA monitoring using c-TCD. Bilateral MCA monitoring may be more advantageous for mild RLS detection.

## Introduction

Recent studies have revealed a complex relationship between RLS and cryptogenic stroke and high-intensity subclinical deep white matter (1). Contrast-enhanced transcranial Doppler is a reliable and reproducible method for detecting RLS with high sensitivity and specificity (2). The Latin American common statement on c-TCD for the diagnosis of RLS recommends monitoring the MCA and mentions that unilateral or bilateral monitoring may result in important bilateral disagreements regarding the outcomes of c-TCD assessments (3). However, at present, there are no reports on c-TCD findings or associations with outcomes of unilateral versus bilateral MCA monitoring. Bilateral MCA monitoring is difficult and time-consuming, and in 10%−20% of stroke patients, the detection of RLS is limited due to insufficient temporal bone penetration (4). The aim of the present study was to observe the difference in the positive rate of unilateral versus bilateral MCA monitoring using c-TCD for the diagnosis of RLS.

## Materials and Methods

### Study Population

We enrolled 239 patients (86 women and 153 men) with a mean age of 48.54 ± 13.25 years (range, 14–79 years), who underwent c-TCD examination in the Department of Transcranial Doppler Ultrasound of our hospital between February 2018 and February 2021, due to suspicion of RLS. The clinical characteristics are shown in Table 1. The 239 patients had bilateral MCA monitoring with dual-channel and dual-depth using c-TCD. The positive rate and shunt classification of RLS monitoring by the left, right and bilateral MCA were counted.

**Table 1:** Demographic and clinical characteristics of the participants.

	**Participants (*n* = 239)**
Age (mean ± SD, years)	48 ± 13.2
Sex (Male/Female)	153/86
**Symptoms or disease**	
Headache	31
Dizziness	18
Ischemic stroke/transient ischemic attack	127/63

Patients with occult stroke, transient ischemic attack, sufficient bilateral temporal window and who were able to complete the Valsalva maneuver (deep inspiration followed by closing the vocal cords and forceful exhalation) were enrolled. Patients with moderate to severe cognitive impairment, poor cardiopulmonary function, and history of PFO closure surgery, and pregnant or lactating women were excluded.

This study was approved by the Ethics Committee of Liaocheng People's Hospital, and all patients provided written informed consent. All procedures in this study were performed according to relevant guidelines and regulations.

### Procedural Technique

#### c-TCD Protocol

The c-TCD examination were performed using a TCD detector (Doppler Box DWL, Germany). Multigate Doppler with standard presets for emboli detection software was used, and the Doppler spectrum and power M-mode were continuously recorded throughout the examination for offline analysis. The patients remained supine and awake throughout the study period. The probe was fixed on the TCD head frame at a frequency of 2-MHz, insonating the bilateral middle cerebral artery at depth of 45–60 mm with a sampling volume of 7–9 mm and a gain of 50–56, while simultaneously displaying the blood flow velocity variation curve and dual M-mode. This procedure was performed under dual-channel and dual-depth conditions.

**Figure 1:**
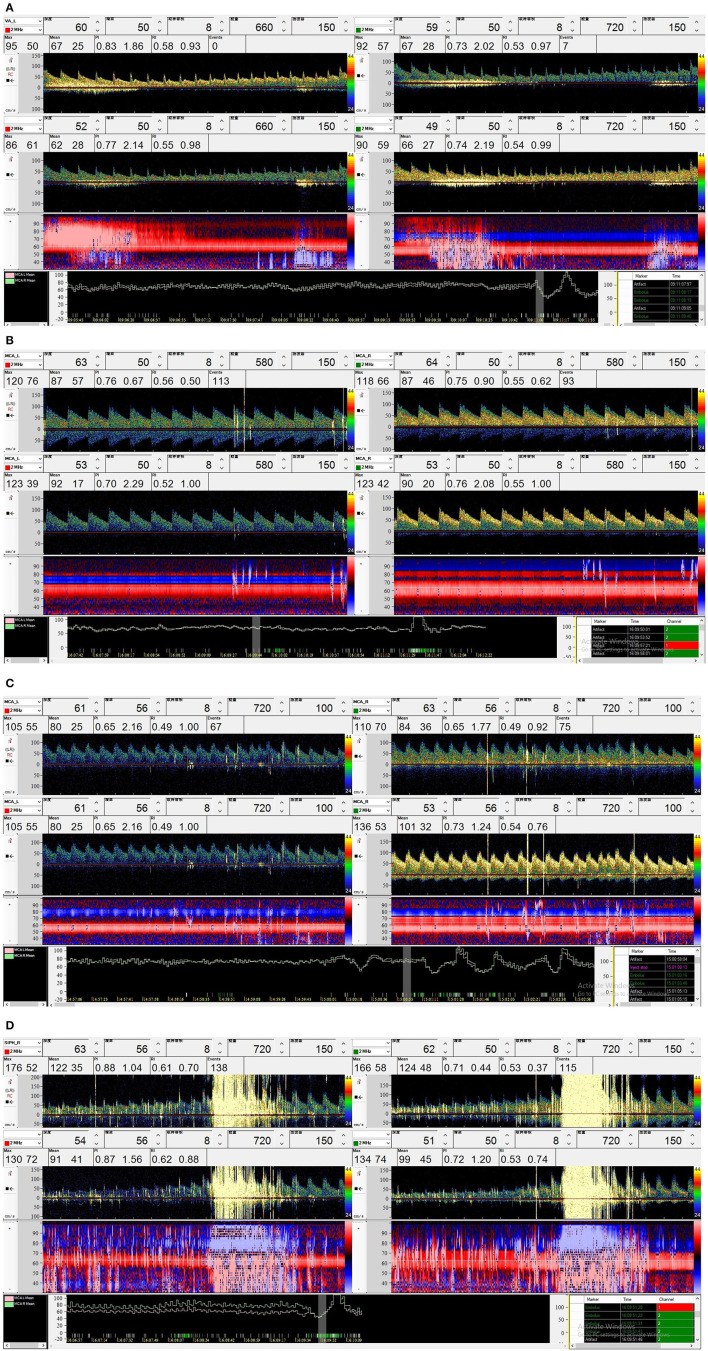
A four-level RLS categorization based on the MB count was applied in our study. **(A)** None (negative result, 0 MBs); **(B)** Mild (1 ≤ MBs ≤ 10); **(C)** Moderate (10 < MBs ≤ 25); **(D)** Large (>25 MBs).

An 18-gauge catheter was placed in the right antecubital vein. The medium was prepared by hand by mixing 9 ml of saline, 1 ml of air, and a drop of the participant's own blood. The medium was rapidly mixed back-and-forth with two 10-ml syringes that were connected by a three-way stopcock 30 times to create microbubbles (MBs). The contrast agent was injected as a rapid bolus. The first injection was performed during normal respiration (rest), and the second injection was performed 5 s prior to the start of a 10-s Valsalva maneuver which reduced the mean MCA velocity by at least 25%. The duration of the Valsalva maneuver was 10 s (3). The presence and number of Mb and the time lag between the injection and the appearance of the first bubble (in seconds) were recorded. All examinations were performed by a single experienced operator (D.R.), who listened to each of the software-recorded signals, watched each signal on the screen, and evaluated the signals offline. Based on the standards reported by Jauss and Zanette (5), a four-level RLS categorization (none, mild, moderate and large) reported by Yang et al. (6) was used in our study. The categorization based on the MB count was applied as follows: none, 0 MBs (negative result, Figure 1A); mild, 1 ≤ MBs ≤ 10 (Figure 1B); moderate, 10 < MBs ≤ 25 (Figure 1C); and large, >25 MBs (Figure 1D). During bilateral monitoring, the highest number obtained in each channel, not the sum of these observations, is considered (3). RLS was considered latent if it occurred only after the Valsalva maneuver and permanent if it also occurred at rest.

### Statistics

The Statistical Program for Social Sciences version 21.0 (SPSS; IBM, West Grove, PA, USA) was used to analyse all data. Differences between groups were analyzed using the *X*^2^ test and the level of significance was set at *P* < 0.05.

## Results

In the LMCA monitoring, 35.56% (85 of 239) of the patients had a positive RLS result, 38 cases were permanent (44.70%), and 47 cases were latent (55.30%). In the RMCA monitoring, 36.82% (88 of 239) of the patients had a positive RLS result, 38 cases were permanent (43.18%), and 50 cases were latent (56.82%). In the bilateral MCA group, 43.09% (103 of 239) of the patients had a positive RLS result, 50 cases were permanent (48.54%) and 53 were latent (51.46%). The positive rate of bilateral MCA monitoring was higher than that of LMCA and RMCA (43.09, 35.56, and 36.82%, respectively), and the difference was not statistically significant (*P* = 0.193).

The positive rate of bilateral MCA monitoring was higher than that of LMCA and RMCA for mild and moderate shunts, but the difference was not statistically significant. The positive rate of bilateral MCA monitoring was equal to that of RMCA but higher than that of LMCA, with no statistical significance (Table 2).

**Table 2:** Positive results of LMC, RMCA, bilateral MCA and shunt degree statistics.

	**RLS (+)**	**Large**	**Moderate**	**Mild**
L-MCA	85 (35.6%)	34 (14.2%)	8 (3.3%)	43 (18 %)
R-MCA	88 (36.8%)	36 (15.1%)	7 (2.9%)	45 (18.8%)
Bilateral MCA	103 (43.1%)	36 (15.1%)	9 (3.8%)	58 (24.3%)
*X* ^2^	3.287	0.089	0.259	3.423
*P-*Value	0.193	0.957	0.879	0.181

LMCA monitoring revealed 85 patients with RLS. The sensitivity was 82.52% (85/103). The specificity was 100%. The positive predictive value was 100%. The negative predictive value was 88.31% (Table 3). The RMCA monitoring results showed 88 cases of RLS. The sensitivity was 85.44% (88/103). The specificity was 100%. The positive predictive value was 100%. The negative predictive value was 90.72% (Table 4).

**Table 3:** Comparison of right-to-left shunt detection by cTCD with LMCA and bilateral MCA monitoring.

	**Bilateral MCA monitoring**	
	**Positive**	**Negative**
**Left MCA monitoring**	
Positive	85	0
Negative	18	136

*Sensitivity 82.52%; specificity 100%; positive predictive value 100%; negative predictive value 88.31%*.

**Table 4:** Comparison of right-to-left shunt detection by cTCD with RMCA and Bilateral MCA monitoring.

	**Bilateral MCA monitoring**	
	**Positive**	**Negative**
**Right MCA monitoring**	
Positive	88	0
Negative	15	136

*Sensitivity 85.44%; specificity 100%; positive predictive value 100%; negative predictive value 90.72%*.

## Discussion

The unclosed foramen ovale (PFO) is the most common type of RLS channel, accounting for approximately 95% of all RLS disorders (7) and other diseases, including patent ductus arteriosus and arteriovenous malformations. Approximately 25% of adults have an incomplete foramen ovale (8). It has been reported in the literature that PFO is significantly associated with cryptogenic stroke, migraine with aura, decompression sickness, and obstructive sleep apnoea (6, 9, 10).

Transesophageal echocardiography (TEE) is considered the gold standard for the diagnosing cardiac RLS. c-TCD has proven to be a sensitive and specific method to diagnose cardiac RLS regardless of location, as documented by several studies published in recent years (11–14). An RLS other than that at the atrial level can only be detected using this method. The advantages of c-TCD include better patient comfort and improved quantitative assessment of the RLS size by combining the use of provocative maneuvers with the ability to identify embolic phenomena in action through real-time monitoring, which has an important functional impact on diagnosis.

PFO may be a risk factor for migraine and ischemic stroke of unknown etiology. The diameters of PFO have ranged from 1 to 19 mm (average 4.9 mm), and PFOs with diameters of 1 to 10 mm accounted for 98% (15, 16). Another autopsy study (17) determined the PFO incidence, and they found that the “probe” patent PFO (0.2–0.5 cm maximum dimension) incidence was 29% and the “pencil” patent PFO (0.6–1.0 cm) incidence was 6%, which reinforced that the smaller PFO accounted for a larger proportion. In our study, both unilateral and bilateral MCA monitoring results showed that the positive rate of mild shunts was higher than that of moderate and large shunts, suggesting that smaller PFOs are more common in patients. In addition, we observed that some mild shunts progressed to moderate RLS, so mild shunts should be taken seriously, and bilateral MCA monitoring is recommended for mild RLS.

In this study, we found that both left and right MCA monitoring have high sensitivity and specificity compared to bilateral MCA monitoring, as measured by the bilateral MCA. Consequently, there was no significant difference between the left - and right-sided monitoring results if only unilateral MCA monitoring was performed when the temporal window was insufficient.

The Latin American common statement on c-TCD for the diagnosis of RLS recommends monitoring the middle cerebral artery bilaterally because the sensitivity of the test and quantification of bubbles may be increased when using simultaneous bilateral monitoring. However, at present, there are no reports on unilateral or bilateral MCA monitoring. In the present study, although there was no significant difference in the positive rate of RLS between bilateral MCA monitoring and unilateral MCA monitoring in 239 patients undergoing c-TCD examination, the results suggest that bilateral MCA monitoring is more likely to improve the positive rate of RLS, especially for mild shunts. This indicates that bilateral MCA monitoring may be advantageous for mild shunts.

Our study has some limitations. First, all patients were monitored in the supine position in the retrospective study, and MCA monitoring in different positions, such as sitting, side decubitus, and standing, was not performed. In addition, the sample size of this study was limited, and further studies with multiple centers and larger samples are needed.

## Conclusion

There was no significant difference in the RLS detection rate between unilateral and bilateral MCA monitoring using c-TCD. Bilateral MCA monitoring may be more advantageous for mild-shunt RLS detection.

## Data Availability Statement

The original contributions presented in the study are included in the article/supplementary material, further inquiries can be directed to the corresponding author/s.

## Ethics Statement

This study was approved by the Ethics Committee of Liaocheng People's Hospital. The patients/participants provided their written informed consent to participate in this study.

## Author Contributions

WZ and KY contributed to conception and design of the study. SW organized the database. HL performed the statistical analysis. LY and WZ wrote the first draft of the manuscript. LW, SW, and KY wrote sections of the manuscript. All authors contributed to manuscript revision, read, and approved the submitted version.

## Conflict of Interest

The authors declare that the research was conducted in the absence of any commercial or financial relationships that could be construed as a potential conflict of interest.

## Publisher's Note

All claims expressed in this article are solely those of the authors and do not necessarily represent those of their affiliated organizations, or those of the publisher, the editors and the reviewers. Any product that may be evaluated in this article, or claim that may be made by its manufacturer, is not guaranteed or endorsed by the publisher.

## References

[1] Dao CN Tobis JM. PFO and paradoxical embolism producing events other than stroke. Catheter Cardiovasc Interv. (2011) 77:903–9. 10.1002/ccd.22884 21207422

[2] Mojadidi MK Roberts SC Winoker JS Romero J Goodman-Meza D Gevorgyan R. Accuracy of transcranial Doppler for the diagnosis of intracardiac right-to-left shunt: a bivariate meta-analysis of prospective studies. JACC Cardiovasc Imaging. (2014) 7:236–50. 10.1016/j.jcmg.2013.12.011 24560213

[3] Zetola VF Lange MC Scavasine VC Bazan R Braga GP Leite ACCB. Latin American Consensus Statement for the use of contrast-enhanced transcranial ultrasound as a diagnostic test for detection of right-to-left shunt. Cerebrovasc Dis. (2019) 48:99–108. 10.1159/000503851 31694010

[4] Seidel G Kaps M Gerriets T. Potential and limitations of transcranial color-coded sonography in stroke patients. Stroke. (1995) 26:2061–6. 10.1161/01.STR.26.11.2061 7482650

[5] Jauss M Zanette E. Detection of right-to-left shunt with ultrasound contrast agent and transcranial Doppler sonography. Cerebrovasc Dis. (2000) 10:490–6. 10.1159/000016119 11070388

[6] Yang Y Guo ZN Wu J Jin H Wang X Xu J. Prevalence and extent of right-to-left shunt in migraine: a survey of 217 Chinese patients. Eur J Neurol. (2012) 19:1367–72. 10.1111/j.1468-1331.2012.03793.x 22747847

[7] Weber F Goriup A. Prevalence of right-to-left shunts in active fighter pilots. Aviat Space Environ Med. (2007) 78:135–6. 17310885

[8] Di Legge S Sallustio F De Marchis E Rossi C Koch G Diomedi M. Short-term and two-year rate of recurrent cerebrovascular events in patients with acute cerebral ischemia of undetermined aetiology, with and without a patent Foramen Ovale. ISRN Neurol. (2011) 2011:959483. 10.5402/2011/959483 22389838 PMC3263533

[9] Schuchlenz HW Weihs W Horner S Quehenberger F. The association between the diameter of a patent foramen ovale and the risk of embolic cerebrovascular events. Am J Med. (2000) 109:456–62. 10.1016/S0002-9343(00)00530-1 11042234

[10] Knauth M Ries S Pohimann S Kerby T Forsting M Daffertshofer M. Cohort study of multiple brain lesions in sport divers: role of a patent foramen ovale. BMJ. (1997) 314:701–5. 10.1136/bmj.314.7082.701 9116544 PMC2126163

[11] Zito C Dattilo G Oreto G Di Bella G Lamari A Iudicello R. Patent foramen ovale: comparison among diagnostic strategies in cryptogenic stroke and migraine. Echocardiography. (2009) 26:495–503. 10.1111/j.1540-8175.2008.00852.x 19452605

[12] González-Alujas T Evangelista A Santamarina E Rubiera M Gómez-Bosch Z Rodríguez-Palomares JF. Diagnosis and quantification of patent foramen ovale. Which is the reference technique? Simultaneous study with transcranial Doppler, transthoracic and transesophageal echocardiography. Rev Esp Cardiol. (2011) 64:133–9. 10.1016/j.rec.2010.10.014 21277667

[13] Martínez-Sánchez P Medina-Báez J Lara-Lara M Oliva-Navarro J Cazorla-García R Ruiz-Ares G. Bajo rendimiento del ecocardiograma, comparado con el Doppler transcraneal, en la detección de la comunicación derecha-izquierda [Low sensitivity of the echocardiograph compared with contrast transcranial Doppler in right-to-left shunt]. Neurologia. (2012) 27:61–7. 10.1016/j.nrl.2011.05.010 21889234

[14] Van H Poommipanit P Shalaby M Gevorgyan R Tseng CH Tobis J. Sensitivity of transcranial Doppler versus intracardiac echocardiography in the detection of right-to-left shunt [published correction appears in JACC Cardiovasc Imaging. 2014 Jan;7:117]. JACC Cardiovasc Imaging. (2010) 3:343–8. 10.1016/j.jcmg.2009.12.012 20394894 PMC3951441

[15] Hagen PT Scholz DG Edwards WD. Incidence and size of patent foramen ovale during the first 10 decades of life: an autopsy study of 965 normal hearts. Mayo Clin Proc. (1984) 59:17–20. 10.1016/S0025-6196(12)60336-X 6694427

[16] Wu CT Han K Guo ZN Yang Y Gao YS Bai J. Effects of patient position on right-to-left shunt detection by contrast transcranial Doppler. Ultrasound Med Biol. (2015) 41:654–8. 10.1016/j.ultrasmedbio.2014.09.005 25683218

[17] Kerut EK Norfleet WT Plotnick GD Giles TD. Patent foramen ovale: a review of associated conditions and the impact of physiological size. J Am Coll Cardiol. (2001) 38:613–23. 10.1016/S0735-1097(01)01427-9 11527606

